# Editorial: Modeling Neurodegeneration in Yeast

**DOI:** 10.3389/fnmol.2021.645190

**Published:** 2021-03-01

**Authors:** Ralf J. Braun, Sabrina Büttner

**Affiliations:** ^1^Research Division for Neurodegenerative Diseases, Center of Biosciences, Danube Private University, Krems an der Donau, Austria; ^2^Department of Molecular Biosciences, The Wenner-Gren Institute, Stockholm University, Stockholm, Sweden; ^3^Institute of Molecular Biosciences, University of Graz, Graz, Austria

**Keywords:** neurodegeneration, yeast, proteopathy, aggregation, cellular fitness

Most age-associated neurodegenerative disorders, including Alzheimer's, Parkinson's, Huntington's, and motoneuron disorders, are characterized by mislocalization, misfolding, and aggregation of disease-specific proteins in distinct neuronal cell populations and are therefore classified as proteopathies (Klaips et al., 2018). While diverse and partially redundant quality control mechanisms are in place to sustain proteostasis and cellular function, the accumulation of aggregation-prone proteins poses a constant burden on the proteostasis systems. With progressing cellular age, different quality control systems functionally decline, and long-lived cells such as neurons are particularly sensitive to misfolding and aggregation of proteotoxic proteins. Disease-associated oligomers and aggregates are directed to distinct protein quality control compartments, thereby compromising overall cellular fitness and survival. Proteopathies are commonly affected by disturbances of protein quality control subroutines, but also by impaired vesicle trafficking and critical mitochondrial damage. For dissecting pivotal interactions among different cellular pathways in the context of proteotoxicity, various cellular models have been established, including the baker's yeast *Saccharomyces cerevisiae* (Braun et al., 2010). *S. cerevisiae* is a genetically amenable unicellular eukaryotic model organism with a high degree of evolutionary conservation, in particular in respect to fundamental cellular processes such as protein quality control pathways, mitochondrial function and vesicle transport. Humanized yeast models based on the expression of human disease-associated aggregation-prone proteins have been successfully used to delineate molecular pathways underlying the loss of cellular fitness. In the Research Topic “*Modeling Neurodegeneration in Yeast,”* we invited researchers to critically summarize recent developments and to present novel data using yeast to study human proteopathies.

The review of Ruetenik and Barrientos provides an overview of the advantages of post-mitotic yeast cultures to model neurodegenerative protein misfolding disorders and summarizes suitable methodology. Thus, this manuscript is of special interest for people new to the field. The reviews from Schneider et al. and Rothe et al. describe the distinct cellular protein quality control compartments in which human disease-associated and aggregation-prone proteins are deposited and discuss why and in which cellular context specific proteotoxic proteins prefer distinct quality control compartments.

The reviews from Hofer et al. and Zheng et al. address yeast models for Huntington's disease (HD), for which many yeast models have been developed so far. The manuscripts give a comprehensive and critical overview of the applied yeast models expressing fragments of the human protein huntingtin, responsible for HD. They describe how these models can be used for pharmacological approaches, and include a mitochondria-associated perspective on Huntington's disease.

Di Gregorio et al., Monahan et al., and Lindström and Liu critically review yeast models for amyotrophic lateral sclerosis (ALS), the most common motoneuron disease. They summarize how yeast ALS models expressing the common ALS-associated proteins TDP-43 and FUS have been successfully used to unravel the molecular mechanisms underlying disease progression.

For Alzheimer's disease (AD), the Research Topic comprises three original studies. One of the pathological hallmarks of AD is the accumulation of the small hydrophobic peptide β-amyloid exerting extra- and intracellular toxicity (Goedert and Spillantini, 2006). Chen et al. and Fruhmann et al. use yeast β-amyloid models to elucidate the intricate interplay between energetics, ER stress and β-amyloid toxicity, with a particular focus on the pathophysiological membrane lesions triggered by β-amyloid. Besides β-amyloid, a mutant variant of ubiquitin, namely UBB^+1^, accumulates in disease-affected neurons in AD. High levels of UBB^+1^ have been described to be detrimental in neurons and yeast cells (Braun et al., 2015). Using yeast expressing UBB^+1^, Muñoz-Arellano et al. challenge this paradigm, showing that UBB^+1^ expression can induce a beneficial stress response. Thus, depending on the cellular context, UBB^+1^ may also enable the cells to cope with proteotoxic stress, which has been confirmed by a more recent study (Verheijen et al., 2020).

For Parkinson's disease (PD), two original studies are included in the Research Topic. Popova et al. provides novel insights on β-synuclein, a homolog of the PD-associated protein α-synuclein. The authors observed that β-synuclein, just as α-synuclein, triggers toxicity in yeast. Of interest, post-translational modification of β-synuclein, namely sumoylation, protects cells from this toxicity. Aufschnaiter et al. worked with another important PD-associated protein, the protein kinase LRRK2. The authors could show that the enzymatic core of this protein compromises mitochondrial function in yeast. While general mitochondrial dysfunction is a well-established hallmark for PD, this study demonstrates that LRRK2 inhibits mitochondrial biogenesis, thus providing a novel perspective on cellular demise during PD.

Finally, Verma et al. could show in their original study that the aggregation of human transthyretin is modulated by yeast prions, thereby confirming the strong interaction of this human protein with the yeast quality control system.

Collectively, this Research Topic highlights the power of yeast models to understand fundamental molecular mechanisms contributing to human proteopathies. We would like to thank all the authors and reviewers contributing to this project. We anticipate numerous novel findings, relevant for human neurodegenerative disorders, based on studies in yeast.

## Author Contributions

All authors listed have made a substantial, direct and intellectual contribution to the work, and approved it for publication.

## Conflict of Interest

The authors declare that the research was conducted in the absence of any commercial or financial relationships that could be construed as a potential conflict of interest.
